# Hybrid Antibacterial and Electro-Conductive Coating for Textiles Based on Cationic Conjugated Polymer

**DOI:** 10.3390/polym12071517

**Published:** 2020-07-08

**Authors:** Natanel Jarach, David Meridor, Marina Buzhor, Daniel Raichman, Hanna Dodiuk, Shmuel Kenig, Elizabeth Amir

**Affiliations:** Department of Polymer Materials Engineering, Shenkar College, 5252626 Ramat-Gan, Israel; nati.j2@gmail.com (N.J.); davidmeridor@gmail.com (D.M.); marinabuzhor@gmail.com (M.B.); daniel.raich1@gmail.com (D.R.); hannad@shenkar.ac.il (H.D.); samkenig@shenkar.ac.il (S.K.)

**Keywords:** antibacterial textile, electro-conductive fabric, coated fabric, polyaniline

## Abstract

The development of efficient synthetic strategies for incorporating antibacterial coatings into textiles for pharma and medical applications is of great interest. This paper describes the preparation of functional nonwoven fabrics coated with polyaniline (PANI) via in situ polymerization of aniline in aqueous solution. The effect of three different monomer concentrations on the level of polyaniline coating on the fibers comprising the fabrics, and its electrical resistivities and antibacterial attributes, were studied. Experimental results indicated that weight gains of 0.7 and 3.0 mg/cm^2^ of PANI were achieved. These levels of coatings led to the reduction of both volume and surface resistivities by several orders of magnitude for PANI-coated polyester-viscose fabrics, i.e., from 10^8^ to 10^5^ (Ω/cm) and from 10^9^ to 10^5^ Ω/square, respectively. Fourier Transform Infrared (FTIR) Spectroscopy and Scanning Electron Microscopy (SEM) confirmed the incorporation of PANI coating with an average thickness of 0.4–1.5 µm, while Thermogravimetric Analysis (TGA) demonstrated the preservation of the thermal stability of the pristine fabrics. The unique molecular structure of PANI, consisting of quaternary ammonium ions under acidic conditions, yielded an antibacterial effect in the modified fabrics. The results revealed that all types of PANI-coated fabrics totally killed *S. aureus* bacteria, while PANI-coated viscose fabrics also demonstrated 100% elimination of *S. epidermidis* bacteria. In addition, PANI-coated, PET-viscose and PET fabrics showed 2.5 log and 5.5 log reductions against *S. epidermidis*, respectively.

## 1. Introduction

During the past decade, functional textiles have received industrial consideration as well as research attention. In particular, fabrics coated with tailored functional materials have been introduced in the cosmetic, pharma, electronic and medicinal fields for therapeutic, controlled release, protecting, sensing, and monitoring applications [1,2,3,4,5,6,7,8,9,10]. Among the different types of functional textiles, antibacterial ones are receiving increasing research and development interest due to health risks associated with infectious bacteria and epidemic outbreaks of viruses throughout the world. Textiles appear in a variety of products in hospitals and homes such as medical clothing, surgery wear, mattress covers, curtains, bedding, and wipes. Consequently, antibacterial coatings for a variety of textiles could reduce the probability of spreading bacteria and viruses in hospitals and homes.

Antibacterial activity is usually attributed to electrostatic interactions between cations and the negatively charged membrane surface of bacteria and/or to other interactions between the cations and the bacteria’s RNA/DNA proteins [11,12,13,14,15,16,17,18,19]. As a result, antibacterial coatings usually contain positively charged materials in the form of either small molecules (mostly metal ions such as copper [11,12,13,17,19,20,21,22], silver [17,18,21], zinc [21,23,24], cobalt [25], nickel [26] or polyoxometalates [27,28]), nanoparticles (such as silver-based nanoparticles [15,29,30,31], rubidium-silver-titanium oxide nanocomposites [32], phenol-based nanoparticles [33] or silica-based nanoparticles [31,34,35,36,37]) or polymeric (such as chitosan/chitin [38,39], modified poly(ethylene imine) [40,41,42,43], quaternary ammonium containing polymers [44,45,46,47,48,49,50], phosphonium containing polymers [49], etc.).

A variety of processes for textile modification with antibacterial coatings have been introduced in recent years. One of the most common methods includes the addition of metal ions to fabrics [51]. Naka et al., for example, registered a US patent for the addition of some metal ions (Ag, Cu, Zn, Al, Mg and Ca) to textiles to obtain an antimicrobial effect [52]. Textile (nonwoven polyester [53,54], nylon [54] and/or cotton [54], woven fabrics such as wool and cotton [55,56] and knitted fabrics [57]) with antibacterial properties were also fabricated by Yamamoto et al. [58], Perelshtein et al. [54], Shah et al. [59] and Erdem et al. [57] using Ag particles. Montazer et al. found that both Ag and TiO_2_ particles could be used for the same purpose [55]. The latter was also proved in a study by Thilagavathi and Parthasarathi [53]. Additional methods such as pad-dry curing [57], spray coating [57], sonochemical coating [54] or the use of nanogels were introduced to improve the adhesion of the particles [56].

Other methods to obtain fabrics with antibacterial properties include the addition of sulfate-containing coatings [60,61,62]. Chitosan and chitosan-derivatives [63,64,65,66,67,68], polypyrrole [69] and ammonium containing silica-particle [70] -treated fabrics have also demonstrated antibacterial properties, and thus, have been studied by several groups. 

**Scheme 1:**
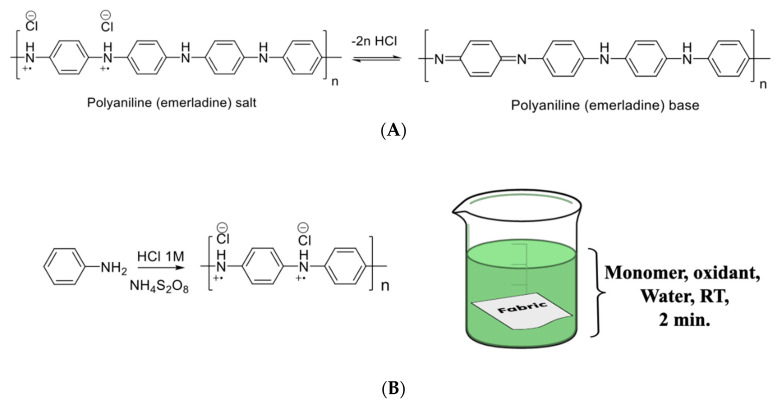
Reversible transformation between polyaniline emeraldine salt and polyaniline emeraldine base (**A**), and polymerization reaction of aniline and schematic representation of fabric coating via in situ polymerization (**B**).

Despite the significant progress that has been achieved in the fabrication of antibacterial fabrics, their industrial realization still requires the study of efficient and green coating processes to ensure uniform coverage with good adhesion and effective concentrations of the active materials. With this goal, we developed a process for grafting nonwoven fabrics with an antibacterial polymer based on polyaniline (PANI) using water as a solvent. Polyaniline is a conjugated organic polymer that contains an amine group in the main chain of each repeating unit. In the emeraldine oxidation state, polyaniline exhibits two distinctive interconverting molecular forms: salt and base (Scheme 1A). Emeraldine base is a neutral form of aniline in which the amine groups are present as imines. Protonation of the imine nitrogen with acid results in the doped emeraldine salt state, in which the imines are converted into quaternary ammonium ions. These ions cause better electron delocalization, converting the nonconductive emeraldine base into a conductive salt form. These unique features, combined with the antibacterial activity of quaternary ammonium ions, make polyaniline a good candidate for the fabrication of antibacterial coatings for textiles. Additional advantages of polyaniline are its good film forming ability and its producibility in water from a low-cost aniline monomer. These characteristics make it possible to coat textiles with polyaniline via aqueous processing techniques. 

Since the pioneering work by Gregory et al. [71], who described the in situ polymerization of pyrrole or aniline on the surface of textile composites, a number of research studies have discussed the production of PANI-coated fabrics and fibers, either by chemical or electrochemical deposition [72,73,74,75,76,77,78,79,80,81,82,83,84,85,86] on polyester (mostly PET) [71,73,74,75,77,78,81,84,85,86], nylon 6 [71,72,75,82,84], wool [75,84], acrylics [75,84] and cotton [75,80,84,87]. The majority of the PANI-coated fabrics and fibers were prepared via in situ oxidative polymerization of aniline under acidic conditions. The polymerization of aniline can be carried out with different oxidation reagents such as potassium iodate [84], ammonium persulfate [50,74,76,84], ammonium peroxydisulphate [72,78,80] and potassium bichromate [86]. Acidic dopants usually include hydrochloric, dodecylbenzene sulfonic and p-toluene sulfonic acids, and the reaction times range between 1–24 h. The properties of PANI-coated fabrics include good electrical conductivity, dissipation of electrostatic charge, electromagnetic behavior, antibacterial properties, material separation and gas sensing.

To the best of our knowledge, the combined effect of antibacterial and electrical conductivity behavior in treated fabrics has not been studied so far. Herein, we describe preparation of PANI-coated nonwoven fabrics via in situ oxidative polymerization of aniline in water (using ammonium persulfate) that can be accomplished in several minutes, producing a uniform and thin coating layer. Three types of nonwoven fabrics, i.e., made of viscose, polyester and viscose/polyester, were examined under different polymerization conditions. In order to evaluate the influence of monomer concentration on the electrical resistivity of the coated fabrics, different concentrations of aniline were employed for the polymerization. The modified fabrics were studied under acidic and basic pH conditions to examine the changes in their electrical resistivity and color at different pH values. Antibacterial properties of the PANI-coated fabrics were evaluated for *S. aureus* and *S. epidermidis* types of bacteria.

## 2. Materials and Methods 

Nonwoven fabrics Viscose (170 g/m^2^, 1.8 ± 0.1 mm thickness and 11–18 µm fiber diameter), Polyester (PET) (150 g/m^2^, 0.6 ± 0.05 mm thickness and 10–23 µm fiber diameter) (supplied by Noam Urim Enterprises Ltd., Kibbutz Urim, Israel) and 50:50 PET:viscose (370 g/m^2^, 0.6 ± 0.07 mm thickness and 9–18 µm of fiber diameter) (supplied by Ofertex, Barkan, Israel) were used. Aniline (99.0% distilled prior to use), ammonium persulfate (98.0%) (Sigma Aldrich, Rehovot, Israel), and hydrochloric acid (HCl, 37.0 wt.%) (purchased from Bio-Lab, Jerusalem, Israel) were used.

Fourier-transform infrared (FTIR) spectra were measured with a Bruker Alpha-P FTIR spectrometer with attenuated total reflectance crystal. The spectra were recorded in the range of 400–4000 cm^−1^ using 2 cm^−1^ resolution and 120 scans.

Scanning Electron Microscopy (SEM) images were taken with the JSM-IT200 microscope (JEOL, Japan). The samples were coated using a SC7620 sputter coater (Quorum, UK). ImageJ software was used to measure the fiber diameters.

The surface and volume resistivities of the coated fabrics were determined (Resistivity test fixture apparatus, KEITHLEY, Tektronix, OH, US). The fabric samples (10 cm × 10 cm) were placed between two electrodes (made of stainless steel) and an electric current was applied (5.0 volt, 0.5 mA). The volume and surface resistivities were between 10^3^ to 10^18^ Ω/cm 10^3^ and 10^17^ Ω/square, respectively.

### 2.1. Coating Procedure 

The polymerization of aniline in the presence of the fabric samples was carried out by a chemical oxidative polymerization process using aniline as a monomer and ammonium persulfate as an oxidizing agent. The nonwoven fabrics (10 cm × 10 cm) were placed into a 1 L Erlenmeyer flask with 200 mL of 1 M hydrochloric acid. Then, aniline was introduced and the fabric was maintained in the solution for 5 min in order to allow absorption of the aniline into the fabric to occur. A solution of ammonium persulfate (1:1.25 molar ratio of aniline: ammonium persulfate) was added dropwise for 5 min at room temperature. At the end of the oxidant addition, the reaction flask was placed into a rotary shaker for two minutes. After completing the coating process, the fabric was washed with water until no color came off it; this was followed by washing with ethanol. The coated fabric was dried under vacuum at 65 °C overnight prior to characterization.

### 2.2. Antibacterial Studies

The antibacterial activity of the various fabrics was evaluated using the Gram-positive bacteria Staphylococcus aureus (*S. aureus*) ATCC 29213 and Staphylococcus epidermidis (*S. epidermidis*) ATCC 1228 as the experimental models. The bacteria were grown overnight in Luria Bertani (LB, Difco, Lapidot, Israel) growth media under shaking (250 rpm) at 37 °C. The following day, the culture was diluted in fresh LB medium by 100 (1%) to achieve 10^5^ colony forming units (CFU) per ml; then, 1 mL from the stock solution was added into each well in a 24-well plate (DE-GROOTH, Danyel Biotech, Israel). Each of the various fabrics was laid on top of the bacterial suspension. The plates were then incubated on a shaker (100 rpm) at 37 °C for 20–24 h. The following day, serial dilutions were carried out and the cells were spotted onto LB agar plates. The LB plates were incubated at 37 °C for 20 h. Cell growth was monitored and determined by viable cell count.

## 3. Results and Discussion

**Figure 1:**
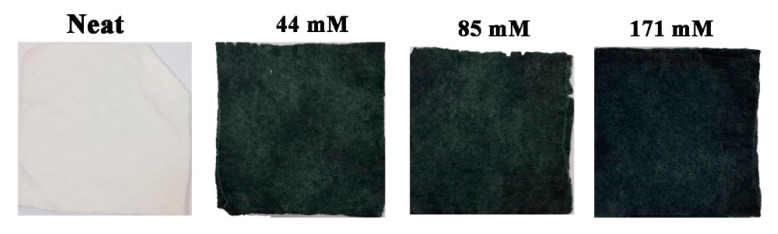
Neat and PANI-coated 50:50 PET:viscose fabrics at different PANI concentrations.

Fabrics (Viscose, PET and 50:50 PET:viscose) were coated with polyaniline via in situ oxidative polymerization, as described above. The aniline concentration was varied (44 mM—low, 85 mM—medium and 171 mM—high) and the molar ratio between aniline and ammonium persulfate was kept at 1:1.25. For all the investigated monomer concentrations, a dark green colored coating was obtained, which became darker with increased aniline concentration (Figure 1 and Figure S1 in the SI). No significant color differences were observed between the three types of fabrics.

**Figure 2:**
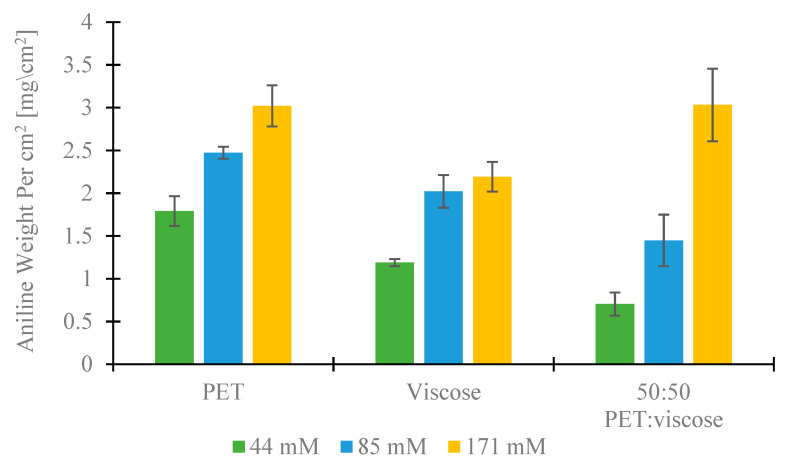
Weight gain of PANI per cm^2^ of fabrics.

By monitoring the changes in fabric weight before and after the process at different aniline concentrations (Figure 2 and Figure S5 in the SI), of the rate-process could be determined. For PET and 50:50 PET:viscose fabrics, the higher the concentration, the greater the percentage of the change in the fabric mass, i.e., increasing the aniline concentration from 44 mM to 85 mM and to 171 mM in PET fabrics led to 7.7 ± 0.7, 10.1 ± 0.2 and 12.8 ± 1.1 % weight gain, respectively, and 2.9 ± 0.6, 5.9 ± 0.9 and 12.8 ± 1.0 % weight gain in the case of PET-viscose coated fabrics. In the case of PANI-coated viscose fabrics, the weight gain increased considerably, i.e., from 6.2 ± 0.4 to 11.4 ± 1.0 % when increasing the monomer concentration from 44 mM to 85 mM respectively. However, no change in weight gain was observed with monomer concentrations of between 85 mM and 171 mM. The effect of the PANI concentration was also measurable in the weight of PANI per cm^2^ of fabric. At low and medium concentrations (44 and 85 mM), PET fabrics gained the highest amount of PANI (1.8 ± 0.2 and 2.5 ± 0.1 mg/cm^2^ compared to 1.2 ± 0.0 and 2.0 ± 0.2 mg/cm^2^, 0.7 ± 0.1 and 1.4 ± 0.3 mg/cm^2^ in viscose and 50:50 PET:viscose, respectively). At the high concentration (171 mM), however, the amount of PANI in PET and 50:50 was almost the same (3.0 ± 0.2 and 3.0 ± 0.4 mg/cm^2^ respectively) while viscose fabrics gained slightly less (2.2 ± 0.2 mg/cm^2^).

Hence, it was observed that the concentration of PANI and the type of fabric influence the amount of polyaniline that is incorporated into the fabric. While the PANI concentration has a greater effect at lower concentrations levels, the fabric chemical nature is dominant at higher ones. When fabric fibers reach monomer absorption saturation, further increases in concentration no longer change the maximum polymer coating weight. Moreover, the faster the fabric reaches saturation, the lower the amount of polyaniline formed. Viscose reached saturation faster than PET and 50:50 fabrics, and thus contained a lower amount of polyaniline coating. As for the effect of the chemical composition, the viscose fabrics contain hydroxyl moieties, while PET contains less hydrophilic ester moieties [88]. Aniline contains primary amine groups that can interact via stronger hydrogen bonding with hydroxy moieties than with esters. Thus, aniline molecules have a lower mobility in viscose than in PET, and therefore, the polymerization rate was slower, manifested as a higher mass of PANI per unit area in PET. The amount of PANI per cm^2^ that was achieved, even at low monomer concentrations, was higher than the required amount found by Varesano et al. to obtain antibacterial properties using polypyrrole coating (>0.0004 gr/cm^2^) [69].

### 3.1. FTIR Analysis 

**Figure 3:**
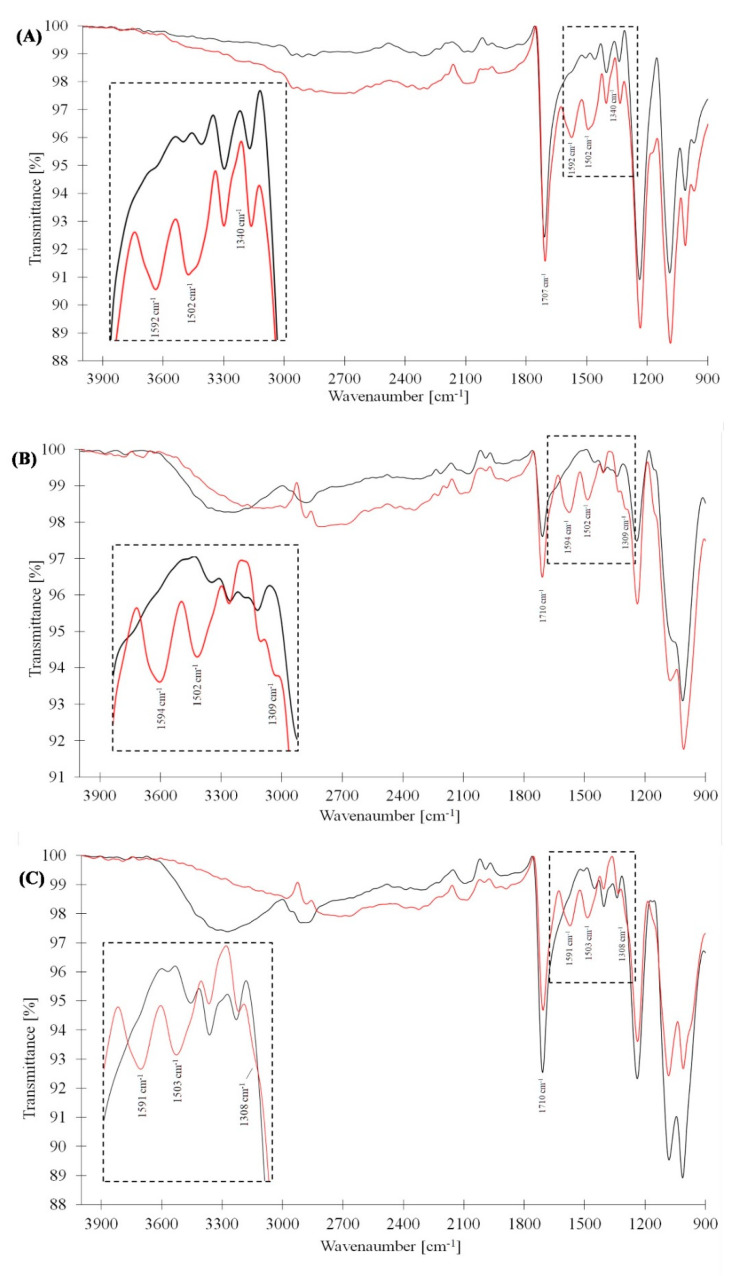
FTIR results of PET fabrics (**A**), viscose (**B**) and 50:50 PET:viscose (**C**). Black line—neat fabrics, and red line—PANI-coated fabrics (171 mM).

The FTIR spectra of PET, viscose and 50:50 PET:viscose emeraldine salt-coated fabrics indicated the presence of polyaniline on the fabric surfaces (Figure 3). The peak at 1308–1340 cm^−1^ refers to the C–N stretching of secondary aromatic amine. The peaks at 1502 and 1592 cm^−1^ were attributed to C=C stretching of the benzenoid and quinoid moieties, respectively, and the peak at 2877 cm^−1^ referred to C–H asymmetrical stretching [89,90]. The broad peak between 3006–3600 cm^−1^ was attributed to O–H stretching in the viscose and N–H stretching in the PANI. It should be noted that due to a natural ageing process that occurred in the viscose fabrics, they (like PET and 50:50 PETviscose fabrics) displayed a small carbonyl peak at 1710 cm^−1^ [91].

In comparing the three different fabrics, it seemed that the effects of PANI were more evident in viscose fabrics, as the C–N peak was most substantial. Between 50:50 PET:viscose and PET fabrics, it seemed that 50:50 PET:viscose had a slightly more significant C–N peak, probably due to the viscose part. Furthermore, following the changes in the OH signals in the 50:50 PET:viscose and viscose fabrics, as shown in Figure 2, 50:50 PET:viscose PANI-coated fabrics exhibited a noticeable reduction in the peak intensity of the OH broad band. In the case of PANI-coated viscose fabric, this reduction was also accompanied by a peak shift, probably due to the difference between conjugated and nonconjugated OH moieties. As with the C–N peak, the changes in the OH were also more observable in 50:50 PET:viscose fabrics than in the viscose ones. 

Unlike the noted differences between neat fabrics and PANI-coated ones, when comparing the ratio between the fabrics’ typical peaks (such as carbonyl) to those of PANI at the three concentrations, no significant differences were detected (Figures S3 and S4 in the SI). This may be due to the lack of FTIR test sensitivity in the case of a low weight, i.e., less than 3.5 mg/cm^2^, PANI coating.

### 3.2. SEM Analysis

**Figure 4:**
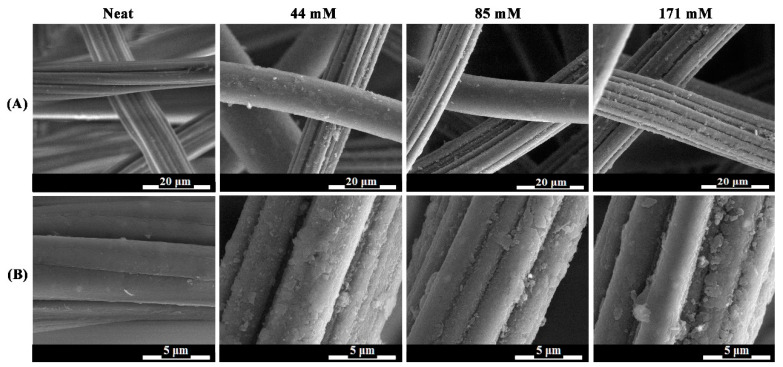
SEM images of 50:50 PET:viscose fabric at 5000× (**A**) and 20,000× (**B**).

As can be seen from the images presented in Figure 4, the modified 50:50 PET:viscose fabrics were clearly coated with PANI. Based on the SEM images, the estimated average coating thickness was between 0.4 and 1.5 µm. These results are in line with data reported in similar studies [71,92,93,94]. However, several differences can be discerned between the three PANI concentrations. When coating the fabrics with 44 mM of PANI, a nonuniform coating layer was obtained, with a low grainsize compared to the other concentrations [93,94]. When the concentration increased to 85 mM, a more uniform and continuous coating was obtained, both on PET and viscose fabrics. Increasing the concentration to 171 mM, however, resulted in a decrease in coating uniformity due to the formation of PANI aggregates, with no change in coating continuity. This may indicate that the optimal concentration is close to 85 mM, or even less, to achieve a uniform continuous PANI coating with a thickness of several hundred nanometers. 

### 3.3. Thermogravimetric Analysis (TGA)

**Figure 5:**
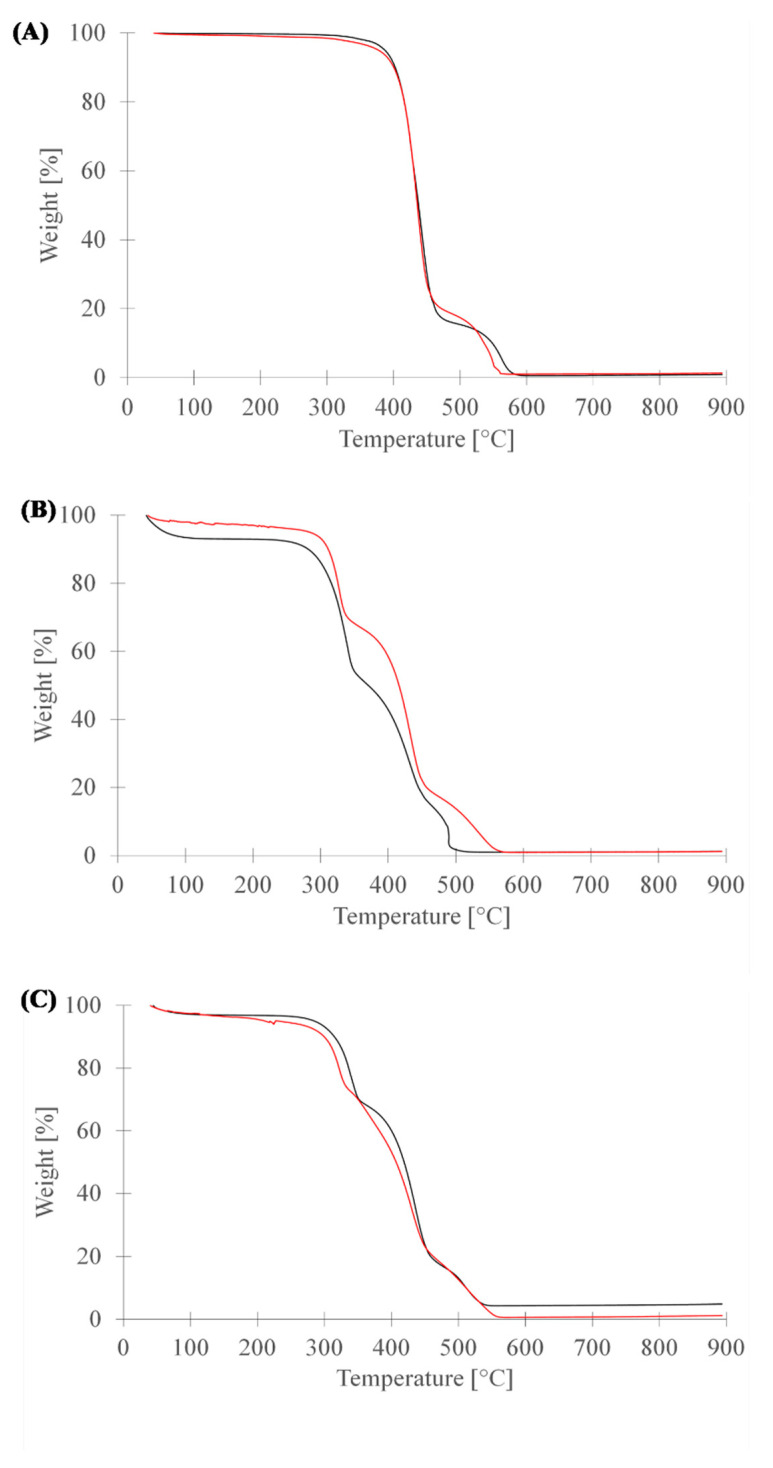
TGA results for neat and PANI-coated PET fabrics (**A**), viscose fabrics (**B**) and 50:50 PET:viscose fabrics (**C**). Black line – neat fabrics and red line—PANI-coated fabrics.

In general, the results of the thermogravimetric analysis (Figure 5) are in line with those reported in the literature [93,94,95,96,97]. The thermal decomposition of the unmodified and PANI-coated PET began at around 350 °C and ended at 450 °C. This decomposition was due to PET polymer backbone degradation [97]. While it seems that the final decomposition of both fabrics occurred within the same temperatures range, PANI-coated PET showed a little more thermal stability, due to the higher stability of the PANI coating. 

Thermal decomposition of the unmodified viscose fabric began at around 230 °C and ended at 330 °C, while for PANI-coated fabrics, it ended at 325 °C (37% weight loss), which was attributed to the destruction of the crystalline structures of the viscose [94] and its amorphous region degradation into D-glucopyranose monomers [91,93,94]. The rest of the coated fabric degradation ended at 548 °C (35% weight loss); this was attributed to the presence of polyaniline emeraldine salt polymer-chains [93,94]. Furthermore, it seems that the coated fabrics were more stable than the neat viscose fabric (exhibiting lower mass loss), probably due to the higher thermal stability of the PANI compared with viscose polymer chains [93]. However, the *T*_max_ (the temperature where the maximum weight loss took place; in this case, it corresponds to the fabric itself) in the coated fabric was equal to those of viscose fabrics, which may indicate that the treatment did not cause any damage to the material.

A comparison of 50:50 PET:viscose fabric with the other fabrics showed that, for the most part, the 50:50 PET:viscose coated fabric was quite similar to viscose fabrics, albeit with slightly lower thermal stability due to the effects of the less stable PET fraction.

### 3.4. Resistivity Measurements

The resistivity measurements revealed that the aniline concentration influences the resistivity of the coated fabrics, as reflected by both the volume and surface resistivity values. When the aniline concentration was increased from 44 to 85 mM, the resistivity decreased until it reached a plateau at 85 mM for both volume and surface resistivities. 

Table 1 shows the average of viscose-polyaniline resistivity (volume and surface respectively) from triplicates samples. The standard deviation of the triplicates shows that the coating process was reproducible and uniform. In addition, the averages of both the volume and surface resistivity indicate that a plateau was reached at an aniline concentration of 85 mM. In comparing the three types of fabrics, it seems that while for the PET and viscose fabrics, the resistivity was lowered by two orders of magnitude relative to the unmodified fabrics, the resistivity of the 50:50 PET:viscose fabric was reduced by four orders of magnitude, and thus displayed the lowest resistivity values. This result is important, since it is challenging to obtain a uniform and interconnected polyaniline network due to the low fiber density of nonwoven fabrics. 

### 3.5. Antibacterial Studies

**Figure 6:**
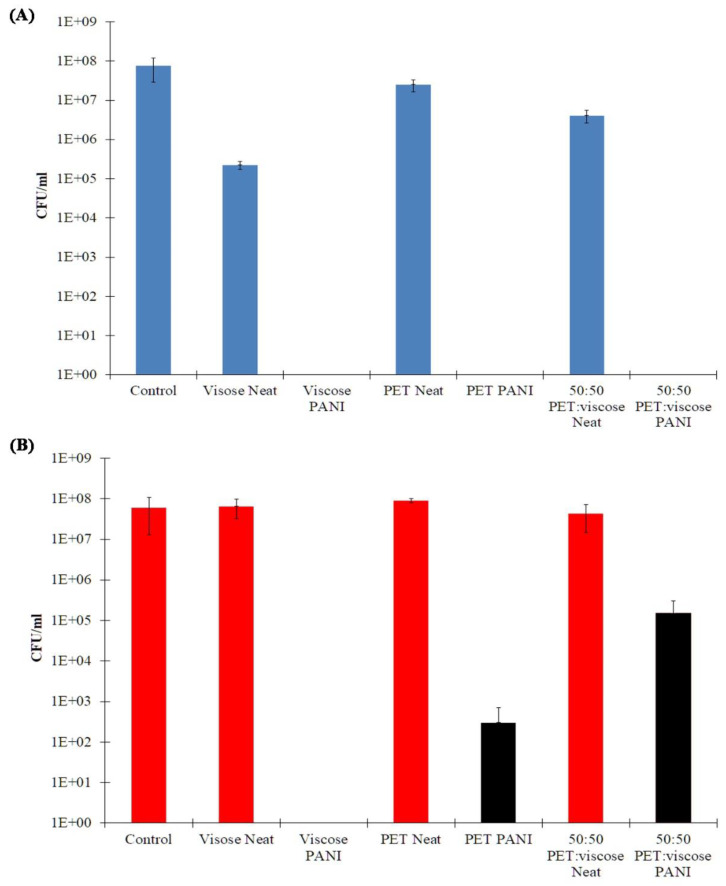
Antibacterial test results of neat and 44 mM PANI-coated viscose, PET and 50:50 PET:viscose fabrics against *S. aureus* (**A**) and *S. epidermidis* (**B**).

In comparing the results obtained for the three untreated fabrics, as shown in Figure 6 and Table S1, it seems that while the uncoated PET and 50:50 PET:viscose fabrics had no inherent antibacterial properties, neat viscose fabrics showed some effect on *S. aureus*. In the literature, this has been attributed to residual chemicals with antimicrobial properties, such as sulfur, remaining from the manufacturing process of these fabrics [98].

The PET, viscose and 50:50 PET:viscose PANI-coated fabrics exhibited a strong antibacterial effect on the *S. aureus* population; the entire population of bacteria from this species did not survive in the presence of the treated fabrics. In addition, PANI-coated viscose fabrics are very effective against *S. epidermidis* (100% reduction, compared to the neat fabrics). On the other hand, PANI-coated PET:viscose and PET fabrics showed a more moderate effect on the population of this type of bacteria, with a 99.65% and 2.5 log and 99.9997% and 5.5 log reduction in the bacteria population, respectively. The differences between the three treated fabrics may be the result of two factors. First, as shown in Figure 2, 50:50 PET:viscose fabrics had the lowest PANI coating weight at 44 mM, and thus, exerted the lowest effect on *S. epidermidis*, which appeared to have greater resistance compared to *S. aureus*. Second, the hydroxyl moieties on the viscose fabrics and/or residual chemicals contributed to the antibacterial effect, and thus, these fabrics showed higher efficiency compared to PET fabrics; 50:50 PET:viscose fabrics contain viscose fibers containing hydroxyl moieties and residual chemicals at half concentration. This, along with the lower PANI coating mass, led to the inhibition of the antibacterial effect against *S. epidermidis*. 

## 4. Conclusions

An efficient, facile and rapid method was developed and studied for coating nonwoven fabrics with polyaniline via in situ polymerization. This process is able to graft a uniform polyaniline coating onto different types of fabrics with various densities and compositions. The method preserves the intrinsic thermal stability of the fabrics. A reduction of both the surface and volume resistivities of several orders of magnitude in comparison with the untreated fabrics was demonstrated. Furthermore, all PANI-coated fabrics displayed excellent antibacterial activity against *S. aureus*, with polyaniline modified viscose fabric also having 100% efficiency against *S. epidermidis*. The combined effect of antibacterial activity and enhanced electrical conductivity suggest that the in situ polymerization of polyaniline is a suitable process for the preparation of functional nonwoven textiles with potential medical applications, due to its inherent cationic and conjugated backbone.

## Figures and Tables

**Table 1:** Resistivity of polyaniline emeraldine salt coated fabrics—(**A**) volume and (**B**) surface resistivity.

**(A)**				
$log\;\left(\frac{\Omega }{\mathrm{cm}}\right)$	**Neat**	**44 mM**	**85 mM**	**171 mM**
50:50 PET:Viscose	8.8 ± 0.9	7.3 ± 0.7	5.8 ± 0.5	5.3 ± 0.5
PET	9.5 ± 0.8	9.0 ± 0.9	8.1 ± 0.8	7.9 ± 0.8
Viscose	8.9 ± 0.7	8.4 ± 0.8	8.0 ± 0.8	7.7 ± 0.8
**(B)**				
$log\;\left(\frac{\Omega }{square}\right)$	**Neat**	**44 mM**	**85 mM**	**171 mM**
50:50 PET:Viscose	9.9 ± 1.0	7.0 ± 0.7	5.8 ± 0.5	5.2 ± 0.5
PES	8.5 ± 0.7	7.9 ± 0.8	7.6 ± 0.8	7.0 ± 0.7
Viscose	9.7 ± 0.9	8.1 ± 0.8	7.9 ± 0.8	7.7 ± 0.8

## Supplementary Materials

The following are available online at https://www.mdpi.com/2073-4360/12/7/1517/s1, Figure S1: Neat and PANI-coated PET (A), viscose (B) and 50:50 PET:viscose (C) fabrics at different PANI concentrations; Figure S2: FTIR of PES fabrics while N- Neat fabrics, L—low concentration (43.7 mM), M—Medium concentration (85.3 mM), H—High concentration (170.6 mM); Figure S3: FTIR of Viscose fabrics while N- Neat fabrics, L—low concentration (43.7 mM), M—Medium concentration (85.3 mM), H—High concentration (170.6 mM); Figure S4: FTIR of 50:50 PES:Viscose fabrics while N- Neat fabrics, L—low concentration (43.7 mM), M—Medium concentration (85.3 mM), H—High concentration (170.6 mM); Figure S5: Weight gain of the PANI-coated fabrics (%); Figure S6: SEM images of neat and coated PET (A), viscose (B) and 50:50 PET:viscose (C) fabrics; Table S1: Percent and log reduction of PANI-coated fabrics against *S. aureus* (A) and *S. epidermidis* (B).
