# Supplementary material for: Hybrid Antibacterial and Electro-Conductive Coating for Textiles Based on Cationic Conjugated Polymer
Source: Polymers (Basel). 2020 Jul 8;12(7):1517. doi: 10.3390/polym12071517 (PMC7407370; doi:10.3390/polym12071517)
Supplement: Supplementary file 1 [file polymers-12-01517-s001.pdf]

Hybrid Antibacterial and Electro-conductive Coating for Textiles Based on Cationic Conjugated Polymer

Natanel Jarach**, David** Meridor**, Marina** Buzhor**, Daniel** Raichman**, Hanna** Dodiuk**, Shmuel** Kenig **and Elizabeth** Amir *

Department of Polymer Materials Engineering, Shenkar College, 5252626 Ramat-Gan, Israel; nati.j2@gmail.com (N.J.); davidmeridor@gmail.com (D.M.); marinabuzhor@gmail.com (M.B.); daniel.raich1@gmail.com (D.R.); hannad@shenkar.ac.il (H.D.); samkenig@shenkar.ac.il (S.K.)

* Correspondence: eamir@shenkar.ac.il

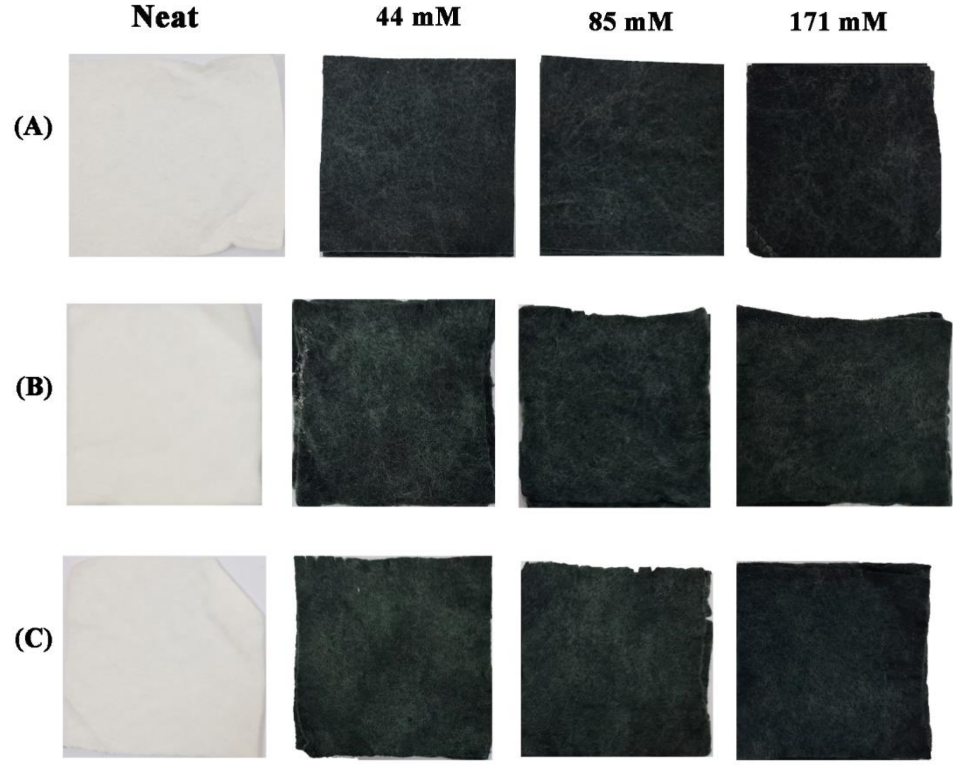

Figure S1. Neat and PANI coated PET (**A**), viscose (**B**) and 50:50 PET:viscose (**C**) fabrics at different PANI concentrations.

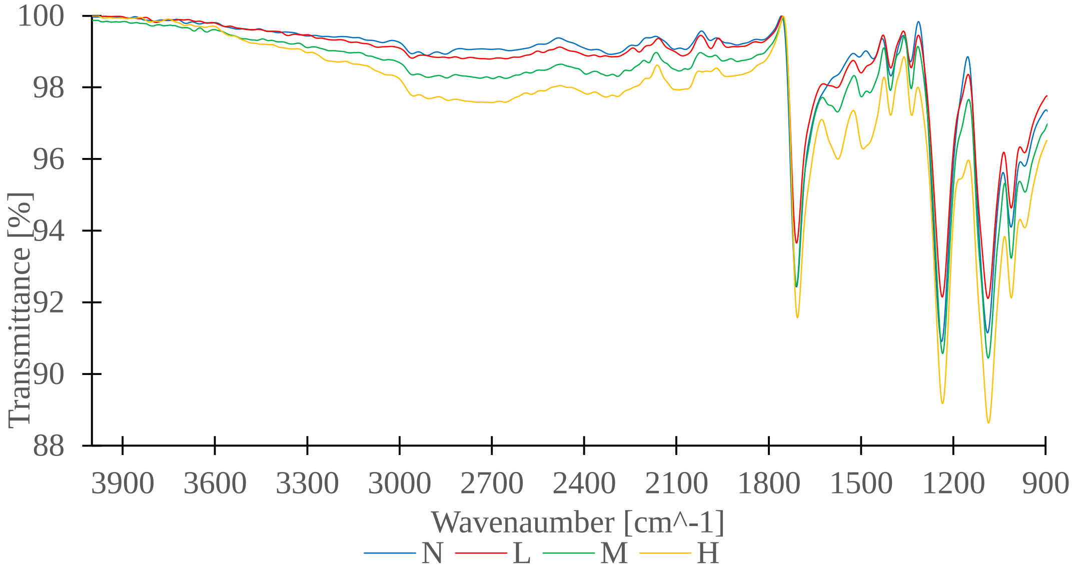

Figure S2. FTIR of PES fabrics while N—Neat fabrics, L—low concentration (43.7 mM), M—Medium concentration (85.3 mM), H—High concentration (170.6 mM).

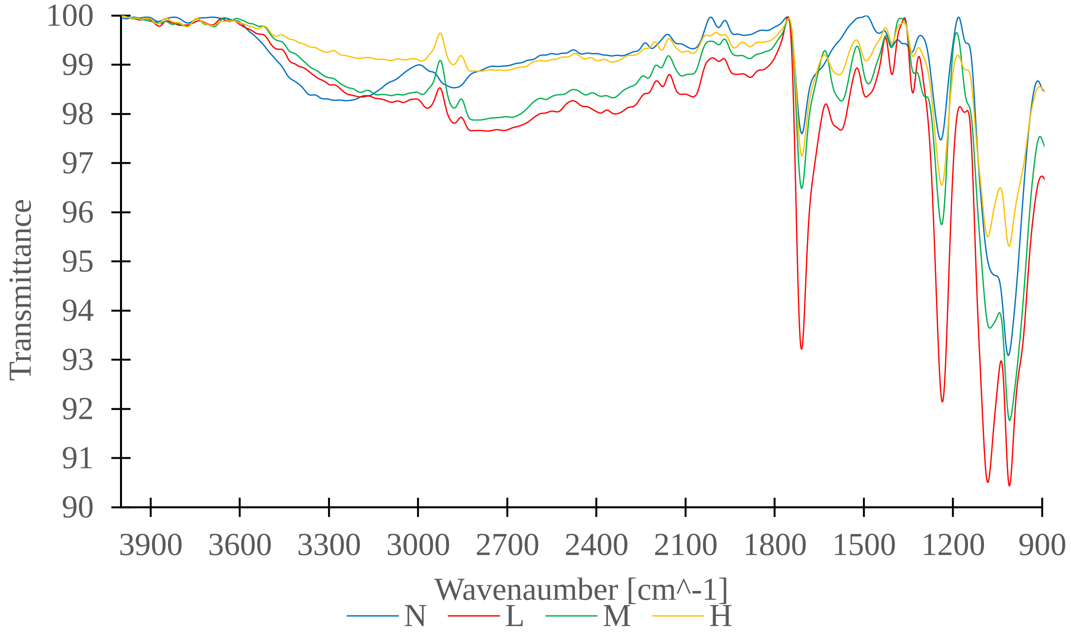

Figure S3. FTIR of Viscose fabrics while N—Neat fabrics, L—low concentration (43.7 mM), M—Medium concentration (85.3 mM), H—High concentration (170.6 mM).

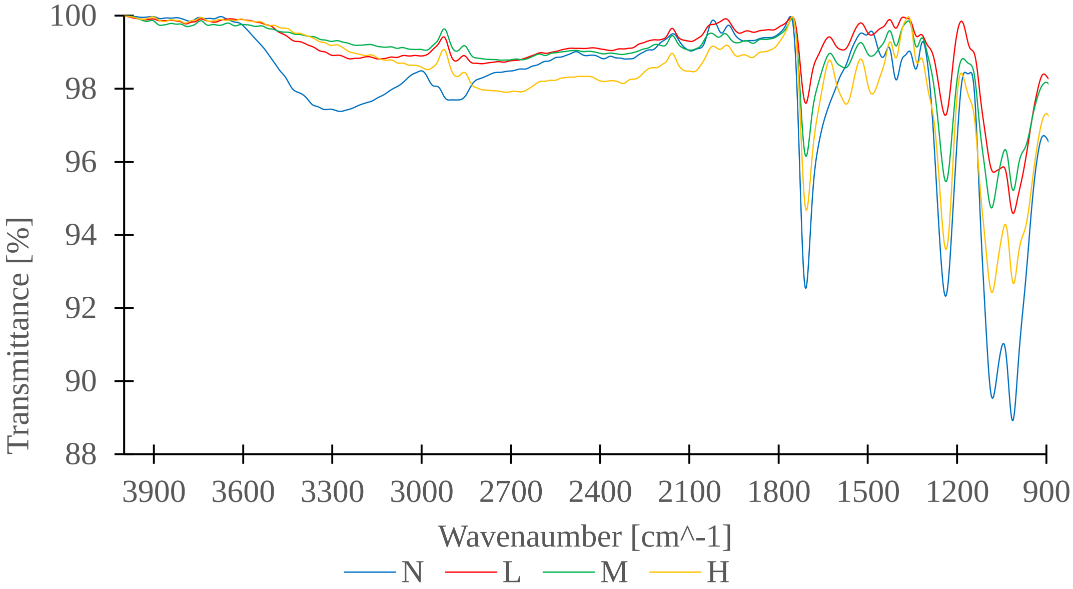

Figure S4. FTIR of 50:50 PES:Viscose fabrics while N—Neat fabrics, L—low concentration (43.7 mM), M—Medium concentration (85.3 mM), H—High concentration (170.6 mM).

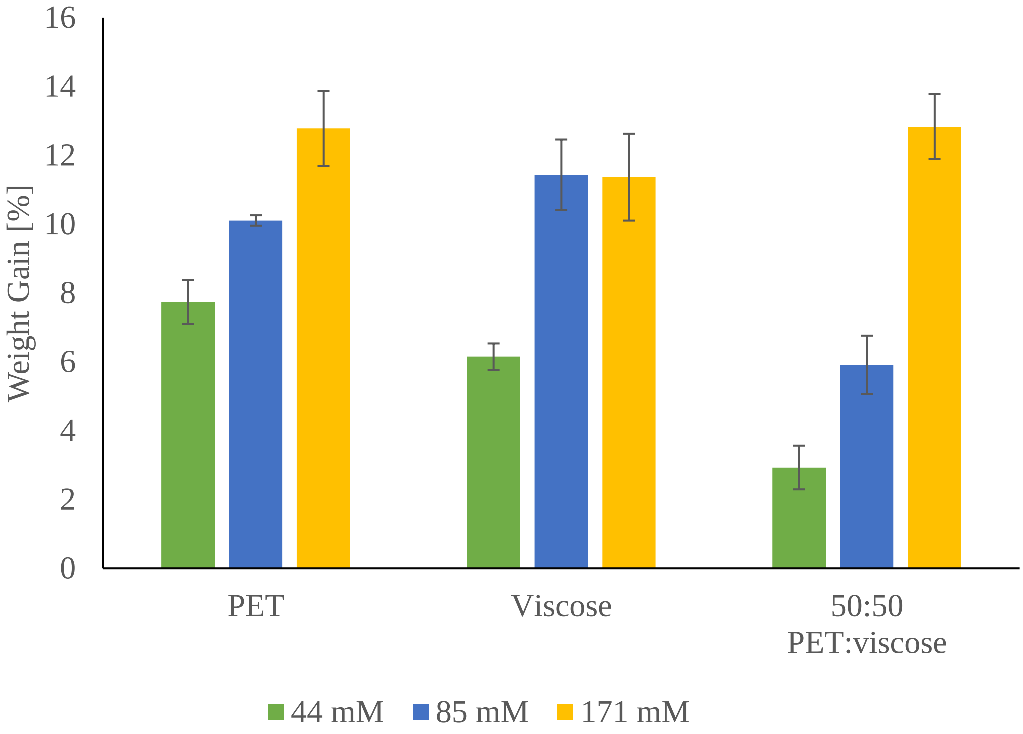

Figure S5. Weight gain of the PANI coated fabrics (%).

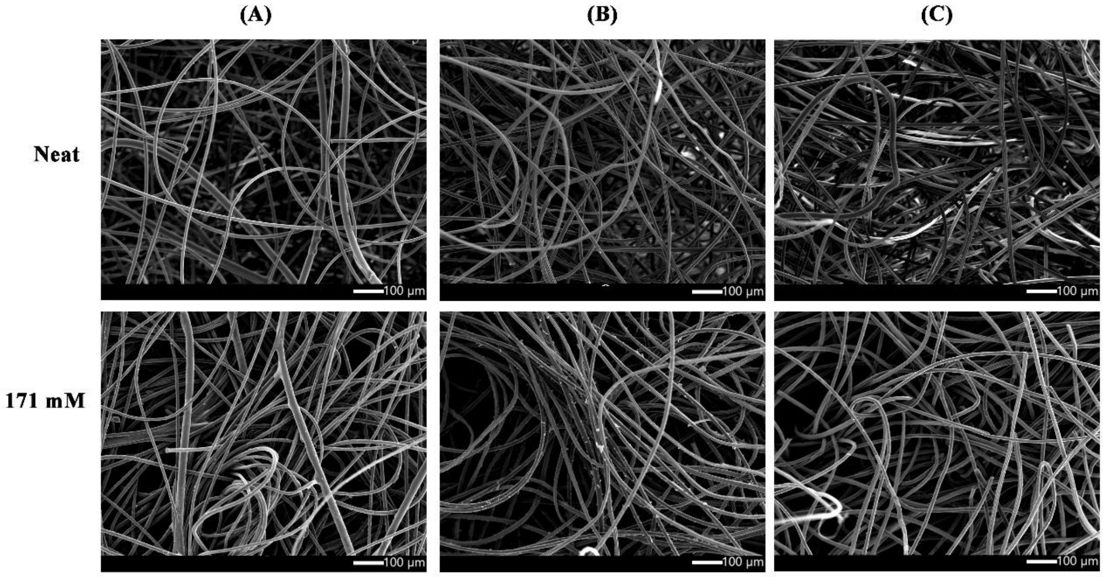

Figure S6. SEM images of neat and coated PET (**A**), viscose (**B**) and 50:50 PET:viscose (**C**) fabrics.

Table 1S. Percent and log reduction of PANI coated fabrics against *S. aureus* (A) and *S. epidermidis* (B).

**(A)**	**Percent Reduction**	**log Reduction**
50:50 PET:Viscose	100%	-
PET	100%	-
Viscose	100%	-

**(B)**	**Percent Reduction**	**log Reduction**
50:50 PET:Viscose	99.6490	2.5
PET	99.9997	5.5
Viscose	100%	-
